# Drone-based entanglement distribution towards mobile quantum networks

**DOI:** 10.1093/nsr/nwz227

**Published:** 2020-01-03

**Authors:** Hua-Ying Liu, Xiao-Hui Tian, Changsheng Gu, Pengfei Fan, Xin Ni, Ran Yang, Ji-Ning Zhang, Mingzhe Hu, Jian Guo, Xun Cao, Xiaopeng Hu, Gang Zhao, Yan-Qing Lu, Yan-Xiao Gong, Zhenda Xie, Shi-Ning Zhu

**Affiliations:** National Laboratory of Solid State Microstructures, School of Electronic Science and Engineering, School of Physics, College of Engineering and Applied Sciences, and Collaborative Innovation Center of Advanced Microstructures, Nanjing University, Nanjing 210093, China

**Keywords:** daytime quantum communication, mobile quantum network, drone, entanglement distribution

## Abstract

Satellites have shown free-space quantum-communication ability; however, they are orbit-limited from full-time all-location coverage. Meanwhile, practical quantum networks require satellite constellations, which are complicated and expensive, whereas the airborne mobile quantum communication may be a practical alternative to offering full-time all-location multi-weather coverage in a cost-effective way. Here, we demonstrate the first mobile entanglement distribution based on drones, realizing multi-weather operation including daytime and rainy nights, with a Clauser-Horne-Shimony-Holt S-parameter measured to be 2.41 ± 0.14 and 2.49 ± 0.06, respectively. Such a system shows unparalleled mobility, flexibility and reconfigurability compared to the existing satellite and fiber-based quantum communication, and reveals its potential to establish a multinode quantum network, with a scalable design using symmetrical lens diameter and single-mode-fiber coupling. All key technologies have been developed to pack quantum nodes into lightweight mobile platforms for local-area coverage, and arouse further technical improvements to establish wide-area quantum networks with high-altitude mobile communication.

## Teaser

We demonstrate the first drone-based entanglement distribution under multi-weather conditions, which exhibits the third possible experimental strategy for quantum communication, in addition to fiber and satellite.

## INTRODUCTION

Quantum communication is the ultimate solution for secure data transferring, where a practical quantum network is expected to establish secure coverage in real time for any locations and sized from local-area to wide-area [1–3]. So far, the most successful quantum networks have been based on the fiber-communication channels and the satellite–ground channels. However, neither of them has fulfilled all the requirements above. Fiber-based quantum communication takes advantage of the well-developed fiber networks for classical communication, whereas it is limited by the availability of the fiber connection [4–8]. The satellite-based free-space quantum communication gets rid of the fixed ground links and it benefits from the lower loss limit in the empty space than the fiber for longer quantum links. Based on comprehensive ground- and aerial-based experimental tests [9–13], a up to 1203-km quantum channel has been built with such quantum satellites [14–18]. However, the existing low-orbit satellite moves in a fixed trace and thus can only establish a quantum data link for certain ground locations within a limited time window. Moreover, such a link has only been established at night so far.

**Figure 1.**
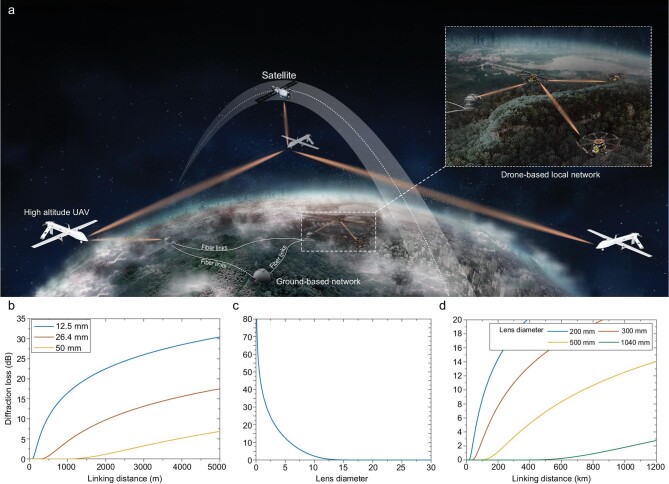
Schematic of a scalable quantum network with drone-based link nodes. (a) Illustrative scheme for a drone-based mobile quantum network from local- to wide-area scales. The local-area network can be established with the plug-and-play drone nodes for fast connection with ground stations, while the wide-area network can be formed by high-altitude UAVs in cascade and connection with existing quantum satellites and ground fiber-based networks. (b) Diffraction loss in a local-area network with small lens diameters that are acceptable on small drones. (c) Diffraction loss as a function of the lens diameter at 100-m link distances, as used in our experiment. (d) Diffraction loss in a wide-area network. Up to 300-km link distances can be expected with reasonable loss and lens diameters using high-altitude UAVs.

To exceed the limits of the existing quantum communication methods, the diverse modern drones may be a good comlementation. The drone, or unmanned aerial vehicle (UAV) [19–21], has undergone an explosive development [22] because of the breakthroughs in the automatic flight-control system and artificial intelligence. It covers a take-off weight from a few grams to tens of tons, a cruising altitude from meters to over 20 km and a flight duration of up to 25 days [23]. Therefore, these various drones can be used to establish a mobile quantum network, for on-demand and real-time coverage at different times and space scales, from local-area networks within kilometers to wide-area networks with hundreds of kilometers and above. As shown in Fig. 1a, both local- and wide-area networks can be expected. In the longer term, such a mobile network could interconnect with satellites and fiber networks for further extension, which will finally form a practical, multifunctional global quantum network.

Here, we realize the first mobile entanglement distribution to connect two ground locations up to 200 meters away. The Clauser-Horne-Shimony-Holt S-parameter exceeds 2.49 ± 0.06 without accidental subtraction. A high-performance airborne entangle-photon source (AEPS) and acquiring, pointing and tracking (APT) systems have been developed and loaded onto home-built octocopters with 35-kg take-off weight and coverage duration of 40 minutes. With uniform lens diameters for all telescopes and single-mode-fiber (SMF) coupling technology, our drone-based

quantum node is highly scalable for further multinode interconnections. This system has proved to be multi-weather compatible through tests during the daytime, clear night and rainy night, with a minimum S-parameter of 2.41 ± 0.24. Our work found the basis for a mobile quantum network and such a network could be built at different scales to achieve full-time all-location multi-weather coverage in future development.

## REASONS TO BUILD A MOBILE NETWORK

Generally, beam diffraction is a fundamental problem for a free-space quantum communication. So far, efforts have been devoted to extending the communication distance in free space, where large lens diameters are required to overcome beam diffraction, such as satellite-to-ground communication. However, free-space communication can be achieved in mobile platforms with much smaller sizes and weights, to achieve plug-and-play local-area coverage. Nowadays, modern picture drones cover a lens diameter from 12.5 mm (DJI Phantom 4 pro, 1375-g take-off weight) to 50 mm (DJI Inspire 2 with Zenmuse X7 gimble camera, 4250-g take-off weight) [24]. In our experiment, we chose a lens diameter of 26.4 mm in this range. Here, we calculate the diffraction-limited link distances with 12.5-, 26.4- and 50-mm lens diameters, which are 180, 802 and 2.8 km at 3-dB loss, respectively, as shown in Fig. 1b and c. With the link distances above, there is no fundamental limit to build a local-area mobile quantum node with a similar size/weight and thus cost in the future, and to establish extensive quantum network coverage in a cost-effective way. In the more distant future, this mobile quantum link may also be used for wide-area-network construction. Being in free space like the satellite–ground link, a lower loss limit can be achieved than in fiber-based links, especially in space-like high-altitude environments. While the diffraction loss can also be significantly reduced through a drone-based quantum network, as we can apply a multinode structure to divide a long link into shorter links. In this case, a reasonable lens diameter can be used to achieve a satisfactory low link loss. This could be a commercial solution for wide-area connection, as the required lens diameter will be smaller for long distances. As shown in Fig. 1d, a 3.0-dB loss can be achieved for a 200-km link with only a 300-mm lens diameter, and such a link distance is within the earth curvature limit at 20-km altitude, where a space-like scattering loss can be achieved in the turbulence-free air.

In this work, we demonstrate the first step towards such a full-scale quantum network with a drone-based entanglement distribution. It is realized with a symmetric transmitter and receiver lens diameters with SMF coupling technology, which makes it scalable for cascaded transmission. This entanglement distribution is achieved over 200 m and a coverage duration of 40 minutes, with a Clauser-Horne-Shimony-Holt (CHSH) S-parameter of up to 2.49 ± 0.06. Multi-weather operations during the daytime, clear night and rainy night are presented because of the high signal-to-noise ratio in our system. Consequently, our drone node is highly reliable, with all-day operation even in a harsh environment. A 12-dB node-to-node loss is achieved with a high-precision closed-loop two-stage airborne APT system, which can be further reduced with better optical alignment. The current quantum drone node is based on an octocopter with a 35-kg take-off weight. A similar system with larger lens diameters can be adapted to high-altitude UAVs towards a multinode network and broad area coverage can be expected.

In the experiment, the main challenge for this drone-based entanglement distribution is to integrate the quantum node into a small drone. The octocopter we developed has a high thrust and low structure weight, and its payloads include an AEPS and two APT units. They are all home-built with a total weight of 11.8 kg, including all the control electronics, which is the key to long flight duration. (For details, see Supplementary Information I).

## AEPS

**Figure 2.**
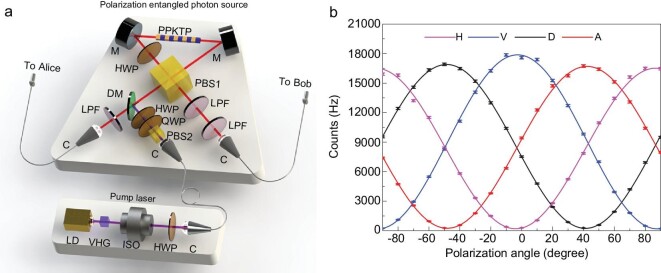
Schematic of the AEPS and its performance. (a) Schematic map of AEPS. The pump laser is a 405-nm LD that is self-injection locked with a volume holography Bragg (VHG) grating. The polarization entanglement is generated in a Sagnac loop with a PPKTP crystal. LD, laser diode; C, collimator; DM, dichroic mirror; HWP, half-wave plate; ISO, isolator; LPF, long-pass filter; M, mirror; PBS, polarization beam splitter; QWP, quarter-wave plate. (b) The measured two-photon correlation in the lab. The interference with one photon projected to $| H \rangle $, $| V \rangle $, $| D \rangle $ and $| A \rangle $ states all have >97.0% visibilities. The error bars represent one standard deviation in the counts.

The AEPS is built with Sagnac interference of two-sided pumped type-II spontaneous parametric down-conversion (SPDC) [25]. The pump light is from a miniaturized single-longitudinal-mode 405-nm laser diode (LD), with a maximum fiber-coupled output power of up to 20 mW before the polarizing beam splitter PBS2. It is based on a Fabry-Perot LD with self-injection locking [26]. The total weight of the AEPS is 468 g and it is sealed in a vibration-isolated package to maintain the performance in the air. The schematic of the Sagnac interferometer is shown in Fig. 2a. The polarization of the laser is controlled by a combination of a half-wave plate (HWP) and a quarter-wave plate (QWP), and focused onto a periodically poled KTIOPO_4_ (PPKTP) crystal at the center of a Sagnac interferometer. By tuning the PPKTP temperature, we achieve degeneration of the SPDC from 405.0 to 810.0 nm (for details, see Supplementary Information II). Focusing is achieved using a homemade collimator that has an aspheric lens to image a beam waist at the center of the PPKTP crystal. The waist size of the pump beam is optimized to achieve high count rates and collection efficiencies into SMFs. The splitting ratio for clockwise and counterclockwise pump power can be optimized to the unit by changing the pump polarization before the dual-wavelength polarizing beam splitter PBS1, so that we can obtain the maximum polarization-entangled-photon state $| \psi \rangle = ({| {HV} \rangle _{12}} - {| {V\!H} \rangle _{12}})/\sqrt 2 $, where $| H \rangle $ ($| V \rangle $) represents the horizontal (vertical) polarization state, and the subscripts 1 and 2 denote two output ports connecting to the Alice and Bob links, respectively. The relative phase change in the distribution links is compensated by a motorized phase shifter formed by wave plates at the output port (for details, see Supplementary Information III). In the lab, under a pump of 15 mW, our AEPS can generate ∼2.4 million entangled-photon pairs per second, based on the coincidence measurement and taking the coupling/detection efficiencies into consideration. This result is comparable to previous work [27] but in a much smaller size. We performed four correlation measurements by projecting the photon in port 1 in $| H \rangle $, $| V \rangle $, $| D \rangle = (| H \rangle + | V \rangle )/\sqrt 2 $ and $| A \rangle = (| H \rangle - | V \rangle )/\sqrt 2 $ states, respectively, and recorded two-fold coincidence counts while changing the linear polarization projection angles in port 2. The visibilities are measured to be 97.4%, 97.7%, 97.1% and 97.0%, respectively, as shown in Fig. 2b. The CHSH Bell inequality is also tested, with the S-parameter measured to be 2.725 ${\rm{ \pm }}$ 0.017.

## APT SYSTEM AND ITS PERFORMANCE

**Figure 3.**
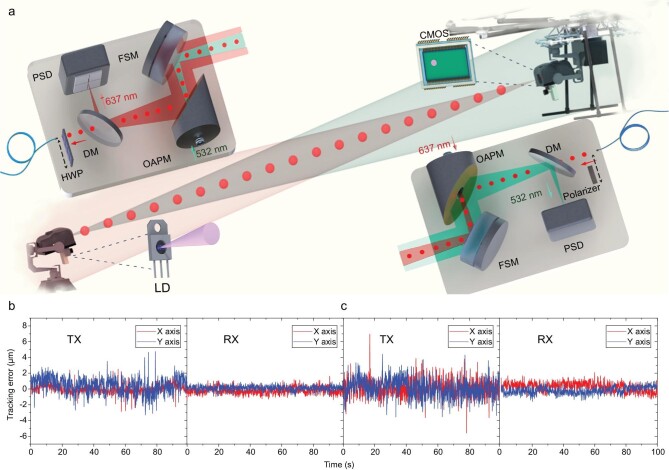
Schematic of the APT system and its performance. (a) Schematic map of the whole APT system. A coaxial zoom camera is used on each APT unit to image the 940-nm LD on the other link side for coarse tracking. It generates the feedback signal to the gimbal stage for moving the image to the center of the CMOS array. The TX and RX telescopes are shown in detail for the two-way fine tracking using the 532- and 637-nm beacon lights. FSM, fast-steering mirror; OAPM, off-axis parabolic mirror; PSD, position-sensitive detector; SMF, single mode fiber. (b) Measured tracking errors of the TX unit and the RX unit before take-off. (c) Measured tracking errors of the TX unit and the RX unit during flight.

The entanglement distribution relies on efficient air-to-ground optical links and multi-weather operation requires SMF collection for both Alice and Bob, which greatly enhances the signal-to-noise ratio but adds more challenges for the tracking precision. Here, it is achieved using a homemade APT system, including two pairs of airborne transmitter APT (TX) units for entanglement-distribution and ground-based receiver APT (RX) units for Alice and Bob stations. These units are designed to realize bidirectional APT [28] for both upward and downward link directions so that both TX units and RX units can be correctly pointed, as shown in Fig. 3a. Each APT unit is composed of a three-axis motorized gimbal stage and a telescope platform. The gimbal stage moves the telescope platform for coarse pointing alignment of the TX/RX telescope by a proportion integration differentiation (PID) error signal calculated from the target image using a coaxial zoom camera. The target for this imaging identification is an uncollimated 940-nm LD on the corresponding receiver or transmitter side. The inset of Fig. 3a shows the zoom-in of the telescope platform. The telescope on each APT unit collimates light to a beam size of 26.4 mm for the 810-nm entangled photons. A carbon-fiber base plate is used for the telescope platform, where the composite structure design is optimized for best thermal stability. For simplicity, a commercially available 50-mm 90-degree off-axis parabolic mirror (OAPM) is used for this collimation. A 637-nm beacon light passes through the central hole of the parabolic mirror for the second-stage fine tracking. The small aperture of this beacon light results in a relatively large divergence angle and thus offers a sufficient field of view for the fine tracking. The fine-tracking loop is also integrated on the telescope platform, which is formed by a position-sensitive detector (PSD) and a fast-steering mirror (FSM). The PSD is mounted at the image position of the transmitter or receiver fiber port to a dichroic mirror (DM). It monitors the focal position of the beacon light to generate the error signal and feeds back to the FSM. With proper feedback electronic controls, the TX unit and the RX unit can be pointed at each other within the accuracy for SMF coupling.

Before the quantum measurement, we perform a classical test of our APT system at a tracking distance of 100 m. The PSD tracking errors are recorded by an onboard data-logging system and typical values at the TX unit and RX unit are shown in Fig. 3b and c. With the octocopter on the ground, the tracking errors are 1.19 ${\rm{\mu m}}$ for the TX unit and 0.57 ${\rm{\mu m}}$ for the RX unit, and they increase to 1.33 ${\rm{\mu m}}$ for the TX unit and 0.62 ${\rm{\mu m}}$ for the RX unit after take-off. All of them are smaller than the mode field diameter of 5 ${\rm{\mu m}}$ for the SMF at 810 nm. An 808-nm LD is used as the reference light and coupled in the transmitter fiber link with a 1% power ratio through a 99:1 fiber coupler. The fiber-to-fiber coupling losses are measured to be around 12 and 14 dB for Alice and Bob, respectively, with negligible weather dependence. The polarization of the reference light is pre-aligned to match the H/V reference frame of the AEPS, and also used for real-time polarization calibration.

The total weight for each TX unit is 3750 g, which is within the payload limit of our octocopter. It cannot be lighter because of the bulky commercial parts such as the OAPM and the PSD, which can be further reduced to fit onto the size of a modern picture drone (for details, see Supplementary Information IV). The coupling loss is mainly attributed to the non-perfect alignment of the telescope, which can also be reduced in the future.

## MULTI-WEATHER ENTANGLEMENT DISTRIBUTION

Between the AEPS and the TX APT units, we have included a real-time polarization correction system to maintain the original entanglement state from the source against in-air vibrations from air turbulence. It offers full polarization control for real-time compensation, under the classical reference from an 808-nm onboard LD. Meanwhile, a classical communication link is utilized for coincident count synchronization from Bob to Alice through a classical optical-fiber communication system. Then our set-up is ready for the entanglement distribution.

For simplicity, we only discuss the link to Alice; the link to Bob is the same. On the receiver side, the RX unit has an adjustable HWP set integrated in the free-space path for the state projection measurement. The collected photons are then coupled into a polarization-maintaining fiber (PMF) and directed to a polarizing beam splitter (PBS) for simultaneous orthogonal polarization projection measurements, with one silicon single-photon avalanche detector (SPAD) for each PBS output. The PBS and the SPADs are both enclosed in a waterproof black box for multi-weather operation. The classical communication from Alice to Bob for coincident count synchronization is achieved through a classical optical-fiber communication system that converts the electrical signal from detectors at Bob to an optical signal and transmits it to Alice through a fiber spool of 300 m. Finally, the received arrival time signals are processed through two time-to-digital converters (TDCs) for coincidence measurements (Supplementary Information V).

**Figure 4.**
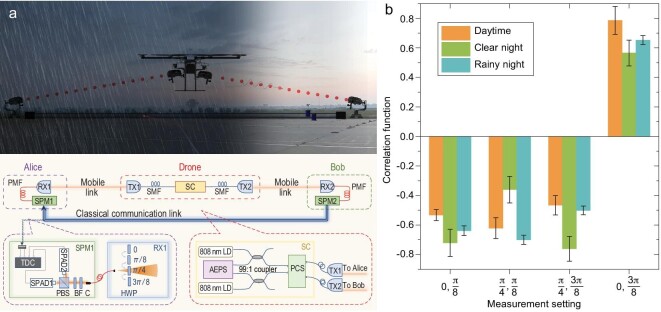
Schematic of the experiments and measurement results. (a) Illustration of the experimental set-up for the entanglement-distribution experiment. The drone node distributes the entangled-photon pairs to the Alice and Bob stations through a mobile link. State projection measurement (SPM) is performed at each ground station and a classical communication link is used for coincidence measurements. BF, band-pass filter; PCS, polarization control system; PMF, polarization-maintaining fiber; SC, source cabin; SPAD, single-photon avalanche detector; TX, transmitter APT; RX, receiver APT; TDC, time-to-digital converter. (b) Measured correlation function results for the CHSH inequality calculation during the daytime, clear night and rainy night. The error bars represent one standard deviation in the counts.

The Alice and Bob ground stations are separated by 200 m, with the octocopter in the center for a single arm length of 100 m for the entanglement distribution. We tested the entanglement state during the daytime, clear night and rainy night, and the experimental scheme is shown in Fig. 4a. The CHSH inequality [29] is used to characterize the entanglement, which is given by

(1) \begin{equation*} S = E(a,{\rm{ }}b) - E(a,{\rm{ }}b^{\prime}) + E(a^{\prime},{\rm{ }}b) + E(a^{\prime},{\rm{ }}b^{\prime}), \end{equation*}

where $a{\rm{ }}(b)$ and $a^{\prime}{\rm{ }}(b^{\prime})$ are the measurement angles at Alice (Bob) and $E(a,{\rm{ }}b)$ etc. are the quantum correlations at the two remote locations. The measurement angles that are changed by the HWP sets inside the RX telescopes are selected among four different groups: ${\rm{(0, 1/8\pi )}}$, ${\rm{(0, 3/8\pi )}}$, ${\rm{(1/4\pi , 1/8\pi )}}$ and${\rm{(1/4\pi , 3/8\pi )}}$. We collected the data for each $E(a,{\rm{ }}b)$ for 120 seconds and got an average count rate of ∼10 Hz. The experimental results are shown in Fig. 4b. The CHSH S-parameters are calculated to be 2.41 ${\rm{ \pm }}$ 0.14, 2.41 ${\rm{ \pm }}$ 0.24 and 2.49 ${\rm{ \pm }}$ 0.06, in the daytime with illuminance of 7316 lx, clear night and rainy night, respectively, without subtracting accidental counts. At all three of the above conditions, the CHSH-type Bell inequalities are violated with up to 8.2 standard deviations, which confirms successful entanglement distribution in our drone-based quantum network.

In this experiment, the octocopter is hovering during the current entanglement distribution. However, we have experienced a maximum wind speed of 18 km/h in the measurement, and the air turbulence can move the airframe around by up to ∼1 m. The high performance of the APT system still offers good tracking for efficient SMF coupling and the real-time polarization-compensation system preserves the state for the Bell-inequality violation. A faster polarization-compensation scheme is under development to achieve entanglement distribution in a high-*g* in-flight maneuver for future applications.

## CONCLUSION

In conclusion, we have shown the first mobile quantum communication via entanglement distribution. Such a system has proved to be robust against sunlight for all-day multi-weather entanglement distribution during the daytime, clear nights and rainy nights, and the S-parameter exceeds 2.49 ± 0.06. We have developed all of the key devices for a lightweight airborne quantum node to fit into a small drone airframe, including a polarization-entanglement source and APT units. A symmetric transmitter and receiver lens diameters are used with SMF coupling, so that the single photons can be collected and retransmitted without a loss in the beam quality. This means that our mobile channel is ready to be scaled towards multinode structures. Therefore, all the key technologies have been presented towards a mobile quantum network. Here, we focus on a local-area network with 40 minutes and 200 m of on-demand coverage. With specially designed components, we may package the whole quantum node into a mini-sized picture drone for a similar local-area network size. Based on this scheme that we have developed, wide-area coverage can be expected by loading the mobile quantum node onto high-altitude UAVs [23]. With reasonable lens diameters within the drone capability and low diffraction loss, long-distance communication can be established within the earth curvature limit at the stratosphere. Such a scalable mobile quantum network has the potential to realize full coverage over multiple space and time scales.

It may not be limited to the entanglement distribution as shown here, Which offers a flexible and cost-effective way for future quantum key distribution [30–32], quantum teleportation [33,34], quantum repeaters [35–38], quantum secure direct communication [39–41] and quantum mechanics foundations testing [42,43]. This network is an important supplement that can link the existing fiber and satellite quantum network, filling the gap in the vast sky in-between and solving mobility and multi-weather-functionality problems. It should also be noted that all the discussions and technologies that we have developed for the scalable multinode network are applicable for all the free-space platforms, including the high-altitude balloon [10], aircraft [44], seawater [45] and satellites [2,14–18].

## Supplementary Material

nwz227_Supplemental_File
