# Supplementary material for: Drone-based entanglement distribution towards mobile quantum networks
Source: Natl Sci Rev. 2020 Jan 3;7(5):921–8. doi: 10.1093/nsr/nwz227 (PMC8446934; doi:10.1093/nsr/nwz227)
Supplement: nwz227_Supplemental_File [file nwz227_supplemental_file.pdf]

Supplementary Information for

Drone-based entanglement distribution towards mobile quantum networks

Hua-Ying Liu*, Xiao-Hui Tian*, Changsheng Gu*, Pengfei Fan*, Xin Ni, Ran Yang, Ji-Ning Zhang, Mingzhe Hu, Jian Guo, Xun Cao, Xiaopeng Hu, Gang Zhao, Yan-Qing Lu, Yan-Xiao Gong[+], Zhenda Xie[+], and Shi-Ning Zhu[+]

National Laboratory of Solid State Microstructures, School of Electronic Science and Engineering, School of Physics, College of Engineering and Applied Sciences, and Collaborative Innovation Center of Advanced Microstructures, Nanjing University, Nanjing 210093, China

I. Payloads on drone.

We fit the payloads under the central deck of the octocopter, in individual compartments for the airborne entangled photon source (AEPS), the two transmitter APT (TX) units and control electronics.

As shown in Fig. S1, the entanglement source cabin (SC) is installed in the central part with shock absorption. Its frame is made of carbon fiber for hosting the 405 nm pump laser, polarization-entangled source, and motorized waveplates for polarization compensation. Both the pump laser and the entanglement source are individually temperature-controlled, and the whole cabin is sealed in a black foam box for further thermal stabilization. We use specially designed rubber absorbers for suspending the whole cabin unit, which reduce mechanical vibrations due to air turbulence.

The two TX units are mounted under the front and rear battery compartments, for head and tail pointing. Each unit is also suspended with 20 rubber absorbers for shock absorption. It works together with the 3-axis gyro built inside the gimbal system for best tracking results.

The two electric compartments (ECs) locate on both sides of the entanglement source cabin, and fit the controllers for fast-steering mirrors (FSM) and position-sensitive detectors (PSD). They also

integrate the power supplies for the coarse/fine pointing beacon light (940 nm/637 nm laser diode (LD)), 405 nm pump laser, 808 nm reference laser, and a number of temperature controllers for the entangled photon source and above LDs.

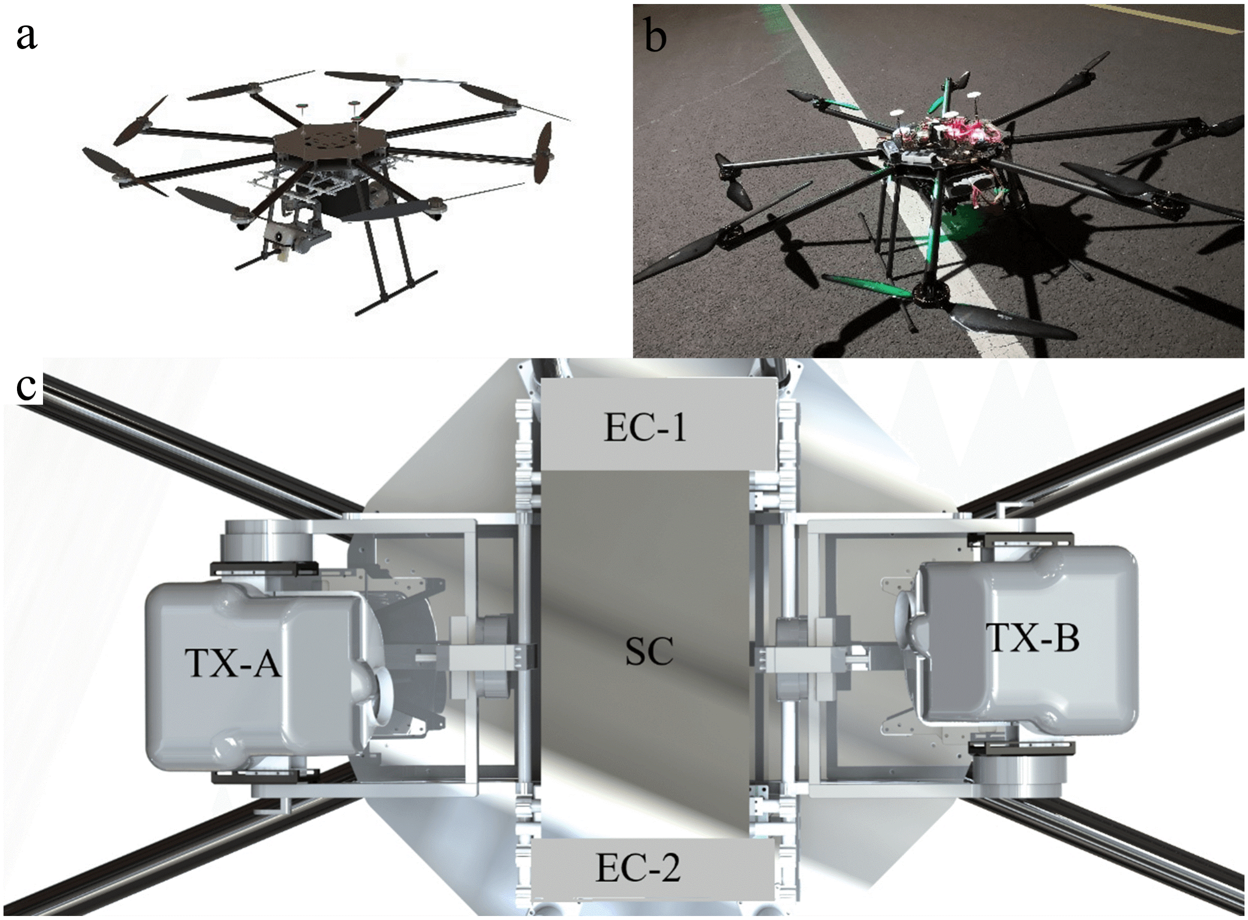

Supplementary Figure S1 | The payloads on drone. a, Schematic of the whole octocopter. The octocopter is composed of eight propeller legs and a central part, with a take-off weight of 35 kg. **b,** The picture of the octocopter. All the equipment is mounted beneath the central deck. **c,** Bottom view of the central deck. TX-A(B): transmitter APT unit for Alice (Bob).

II. Phase-matching measurement for the entanglement source

The spectrum of the AEPS is determined by the phase-matching condition of the spontaneous parametric down-conversion (SPDC) process. In order to get a maximum generation rate and collection efficiency, we choose type-II degenerate SPDC progress. Here it is achieved by finely

tuning the periodically poled $KTiOPO_4$ (PPKTP) temperature while the pump laser is fixed at 405.0 nm. The poling period of the PPKTP crystal is 10.025 μm, and we can calculate the phase-matching curve using the Sellmeier equation of the KTP crystal[1, 2], as shown in Fig. S2a. The frequency degeneracy can be achieved at 50.6 °C. In experiment, the single photon spectrum of the SPDC process is captured by a grating spectrometer with a high-sensitivity CCD camera. Fig. S2b shows the SPDC spectra at PPKTP temperature of 21.9 °C and 18.4 °C. At 21.9 °C the signal and idler photons are nondegenerate with 1.7 nm separation, and the full width half maximum (FWHM) is measured to be 0.4 nm. The narrow peak in between them is the second-order diffraction of the residue pump. The right figure of Fig. S2b shows the SPDC spectrum at 18.4 °C, where frequency degeneracy can be achieved. The difference in the phase-matching temperature between experiment and simulation results from a small deviation of poling period.

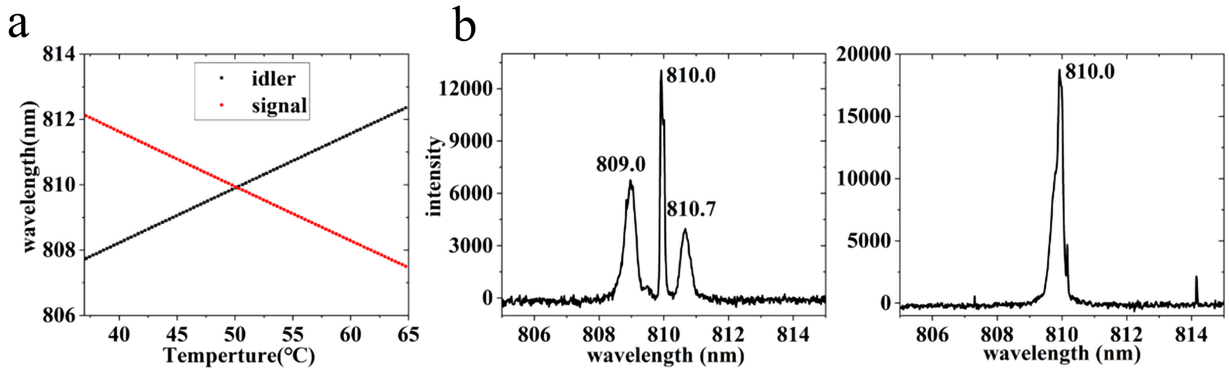

Supplementary Figure S2 | Phase-matching for the photon source. a, Calculated tuning curve for the signal and idler wavelength as a function of PPKTP temperature. **b,** SPDC spectra captured at 21.9 °C (left) and 18.4 °C (right). The narrow peak at 810.0 nm is the second-order diffraction of the residue pump light.

III. Details of the pump source and polarization compensation

We use a self-injection-locked Fabry-Perot LD as pump. As shown in Fig. S3b, the whole pump unit is integrated on a monolithic aluminum baseplate with temperature control. The light from the laser chip is partially reflected back by a narrow-linewidth volume holographic grating (VHG).

This external feedback injection-locks the LD so that the single-longitudinal-mode lasing can be achieved. The pump wavelength is measured to be 405.0 nm. An optical isolator is used to suppress any unwanted reflection feedback. Then the pump light is coupled into a polarization-maintaining fiber (PMF) and directed to polarization-entangled photon source on the left side.

There are fiber cables and other birefringent components between the AEPS and the receiver APT (RX) unit, which may change photon polarization. Moreover, the fiber cables between the entanglement source cabin and the TX telescope can be twisted and change the polarization when the octocopter is in motion. Therefore, we have to integrate a polarization compensation system in the entanglement source cabin on the octocopter, for real-time polarization compensation in quantum links. Such compensation is achieved by the motion of a number of motorized waveplates with the assistance of an 808 nm LD. This laser is remotely controlled by an electronic switch, and polarization compensation is performed when the laser is on.

For an arbitrary polarization entanglement state, the full polarization compensation can be achieved in two steps: 1, matching the polarization axes between the AEPS and the RX unit; 2, correcting the relative phase between $|HV\rangle$ and $|VH\rangle$ in the Alice or Bob link. The polarization compensation geometry is illustrated in Fig. S3a. We use fiber U-Benches to access free-space optical path for this polarization compensation using a group of waveplates. For simplicity, here we only discuss the compensation process in the Alice link, and it is similar in the Bob link except for the absence of the relative phase compensation between $|HV\rangle$ and $|VH\rangle$ part. A 99:1 fiber coupler (coupler1) is inserted in the fiber link between the AEPS and the TX unit. The entangled photons are directed for 99% transmission, while the 808 nm laser light is injected through the 1% port of coupler1. The polarization of the laser is pre-aligned to match the $|H\rangle$ polarization of the AEPS.

U-Bench1 is used for the polarization axes matching between AEPS and RX unit, where a half-wave plate (HWP1) and a quarter-wave plate (QWP1) offer full polarization control in this link. At a ground station, we can switch the output from state-projection PBS to either a single photon

avalanche detector (SPAD) or a power meter. When the power meter is switched in, we monitor the output power, and scan the angles of HWP1 and QWP1 for power cancellation. A high power rejection can be reached showing a good polarization axes match, and this process can be performed via remote control for a real-time correction.

U-Bench3 controls the relative phase between H and V polarizations in the Alice arm and consequently the phase difference between the $|HV\rangle$ and $|VH\rangle$ components in the entanglement state. U-Bench3 consists of two 45°-oriented quarter-wave plates QWP3 & 4 with a motorized HWP3 in between. By changing the angle of HWP3 the relative phase can be tuned independently without affecting the H and V polarization orientation.

The entanglement state we plan to distribute is $|\psi\rangle = |HV\rangle_{12} - |VH\rangle_{12}$, which results in a coincidence cancelation in $|DD\rangle$ state projection. We rotate HWP3 in U-Bench3 while monitoring the coincidence counts in the $|DD\rangle$ state projection. The phase compensation is finished when the optimized cancelation is observed. We only need to perform this phase compensation before takeoff, as the fiber bending during flight can be fully compensated by only polarization axes matching.

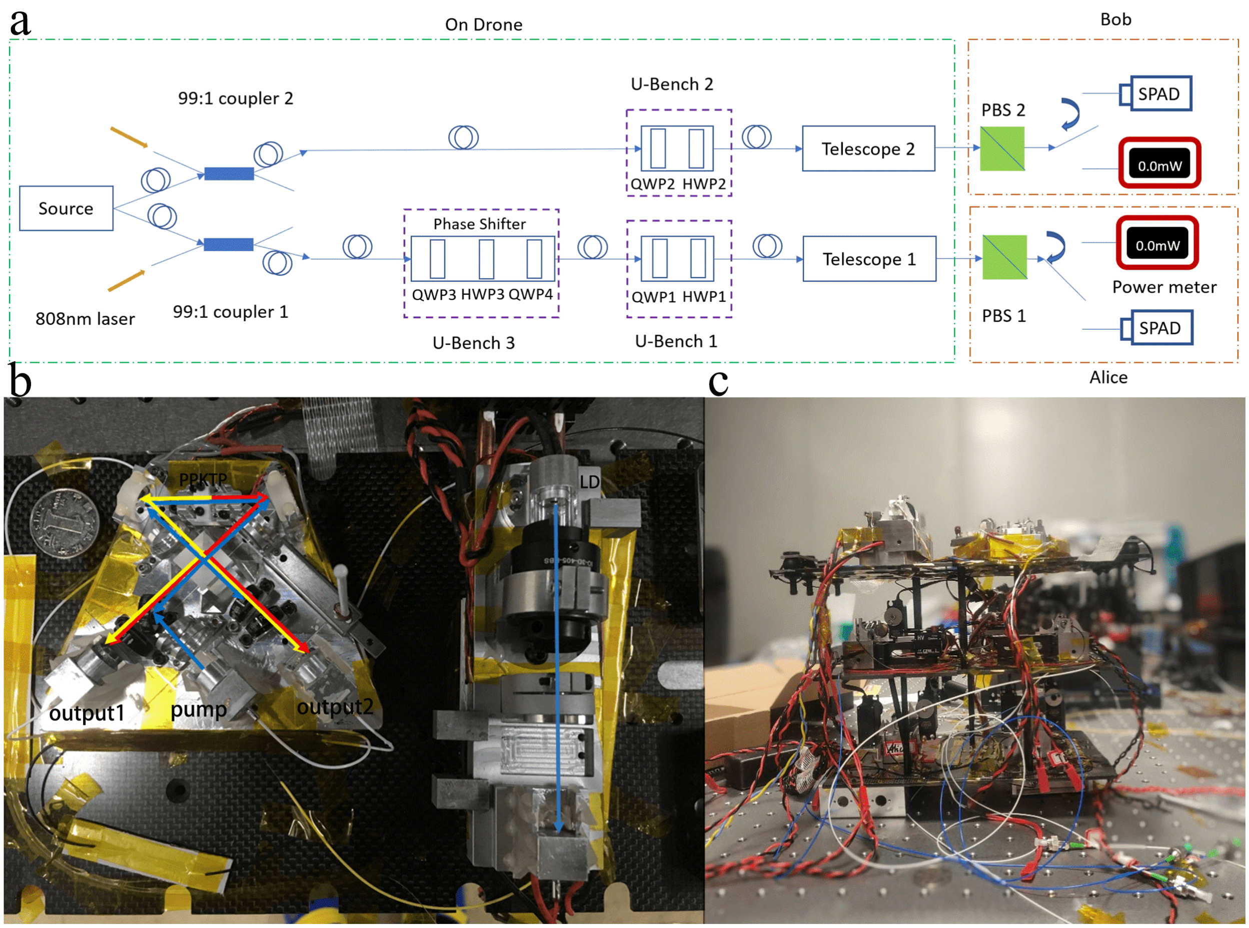

Supplementary Figure S3 | Schematic of polarization compensation progress and entanglement source cabin details. a, Schematic of polarization compensation progress. Two 99:1 couplers are used to couple the laser light for polarization compensation. U-Bench1 and 2 are used for real-time polarization axes matching for Alice and Bob, respectively, while U-Bench3 is used for the phase compensation. **b,** Top view of the entanglement source module with the pump laser. It is mounted in the top floor inside the SC. **c,** Side view of the SC. The lower floors sit the polarization compensation system.

IV. APT system performance test

Our airborne TX and ground-based RX telescopes have the same beam aperture, and share the same gimbal stage, telescope baseplate and the enclosure design. Their appearance is shown in Fig. S4a and b, respectively. The gimbal stage is powered by three brushless motors for the angular

motion on pitch yaw roll axes. No gears are used in this direct drive setup for backlash-free operation. A 3-axis gyroscope chip is mounted on the telescope baseplate for the active stabilization. We use enclosures to cover the telescopes for water protection and blocking the background light. On the RX unit, a long black carbon fiber tube is added as angular aperture for further background rejection. With the two-stage APT using the gimbal stage and fast steering mirror (FSM) in close-loop with corresponding position-sensitive detector (PSD), the quantum link can be established reliably. Detailed structures and performance of the system is shown in Table S1.

We use commercial-available parts in our current APT unit, which prevent the further reduction in the size and weight. For example, as shown in Fig. 1b, an 11.5 mm beam aperture is sufficient for this local-area network connection in 200 meters, and a much smaller collimating mirror can be used to replace the current one with 50.8 mm in diameter. Other components like the PSD and FSM can also be specially designed for this application. In fact, a DJI Phantom 4 picture drone has a similar camera lens diameter of 12.5 mm, and its take-off weight is only 1.4 kg. With proper components and design, there is no fundamental limit to adapt the quantum node to a picture drone size.

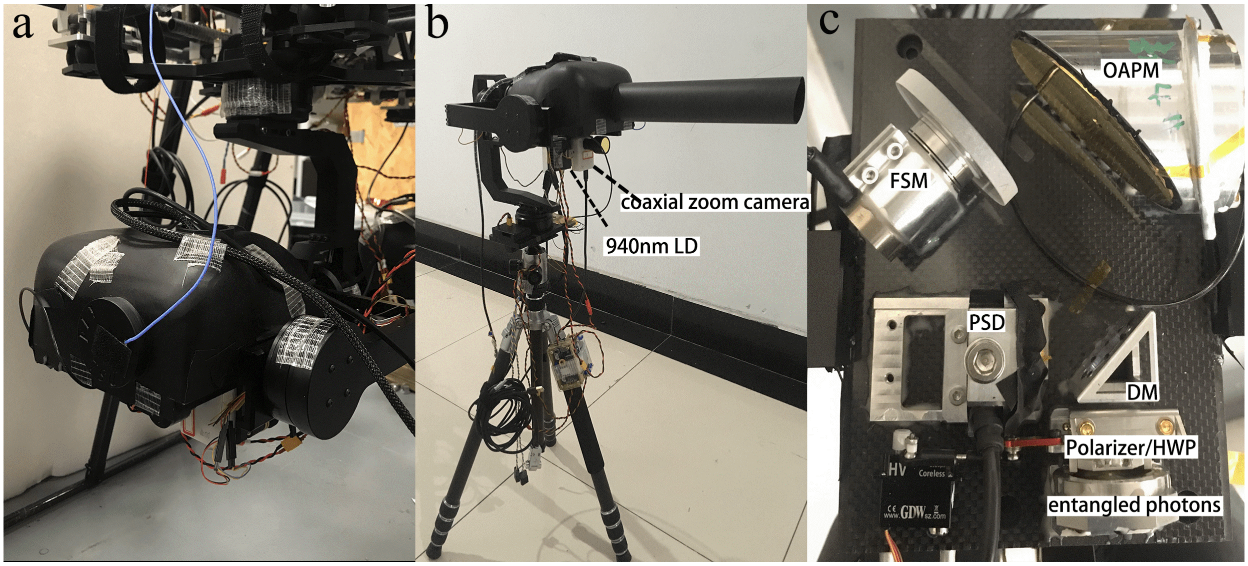

Supplementary Figure S4 | Pictures of APT units. a, A picture of TX unit. The telescope

platform is sealed in an enclosure to avoid direct exposure to dirt, rain, and background light. **b,** A picture of the RX unit. **c,** A picture of an APT telescope.

**Performance of the APT system**			
**Components**		**Transmitter**	**Receiver**
Coarse pointing mechanism	Type	3-axis motorized gimbal stage	3-axis motorized gimbal stage
	Tracking range	Yaw：± 45 °<br>Pitch：± 15 °<br>（With Roll fixed）	Yaw：± 45 °<br>Pitch：± 15°<br>（With Roll fixed）
Coarse pointing camera	Type	COMS	COMS
	FOV	0.11 rad × 0.08 rad	0.11 rad × 0.08 rad
	Size & Frame rate	640 × 480 pixels & 60 Hz	640 × 480 pixels & 60 Hz
Coarse pointing beacon laser	Power	2 W	2 W
	Wavelength	940 nm	940 nm
	Divergence	0.35 rad × 0.07 rad	0.35 rad × 0.07 rad
Fine tracking mechanism	Type	PZT FSM	PZT FSM
	Range	± 1.75 mrad	± 1.75 mrad
Fine tracking sensor	Type	PSD	PSD
	FOV	40 mrad × 40 mrad	40 mrad × 40 mrad
	Size & Frame rate	4 mm × 4 mm & 60 kHz	4 mm × 4 mm & 60 kHz
Fine tracking	Power	30 mW	70 mW
	Wavelength	532 nm	637 nm

beacon laser	Divergence	10 mrad	10 mrad
Tracking error		1.15 μm × 1.33 μm	0.62 μm × 0.46 μm

Supplementary Table S1. Performance of the APT system.

V. Ground station

Our ground setup is composed of Alice and Bob ground stations and a two-channel classical communication link in between. Each ground station includes a RX unit and Bell-test module with their control electronics. Fig. S5 shows pictures of the ground stations under different weather conditions. When a photon is captured by the RX unit, it is directed through a PMF to the Bell-test module for projection measurement. Two single-photon avalanche detectors (SPADs) are used to detect photons from two outcomes of the state projection at each ground station. All detector clicks from Alice and Bob stations are registered for four coincidence measurements simultaneously.

As shown in Fig. S6, a two-channel fiber optical classical communication link is used to transmit the SPAD clicks at Bob station to Alice, so that the coincidence measurement can be performed at Alice station. Each communication channel is connected to one Bob's SPAD, and the electric avalanche pulses are converted to a classical optical pulse via an optical transmitter unit. A 300-meter fiber spool is used to transmit the optical pulses to Alice station in each channel, and the pluses are converted back to electrical pulses by the optical receiver unit. Therefore we can make coincidence measurements with the Alice's photon clicks with two time-to-digital converter modules (TDC).

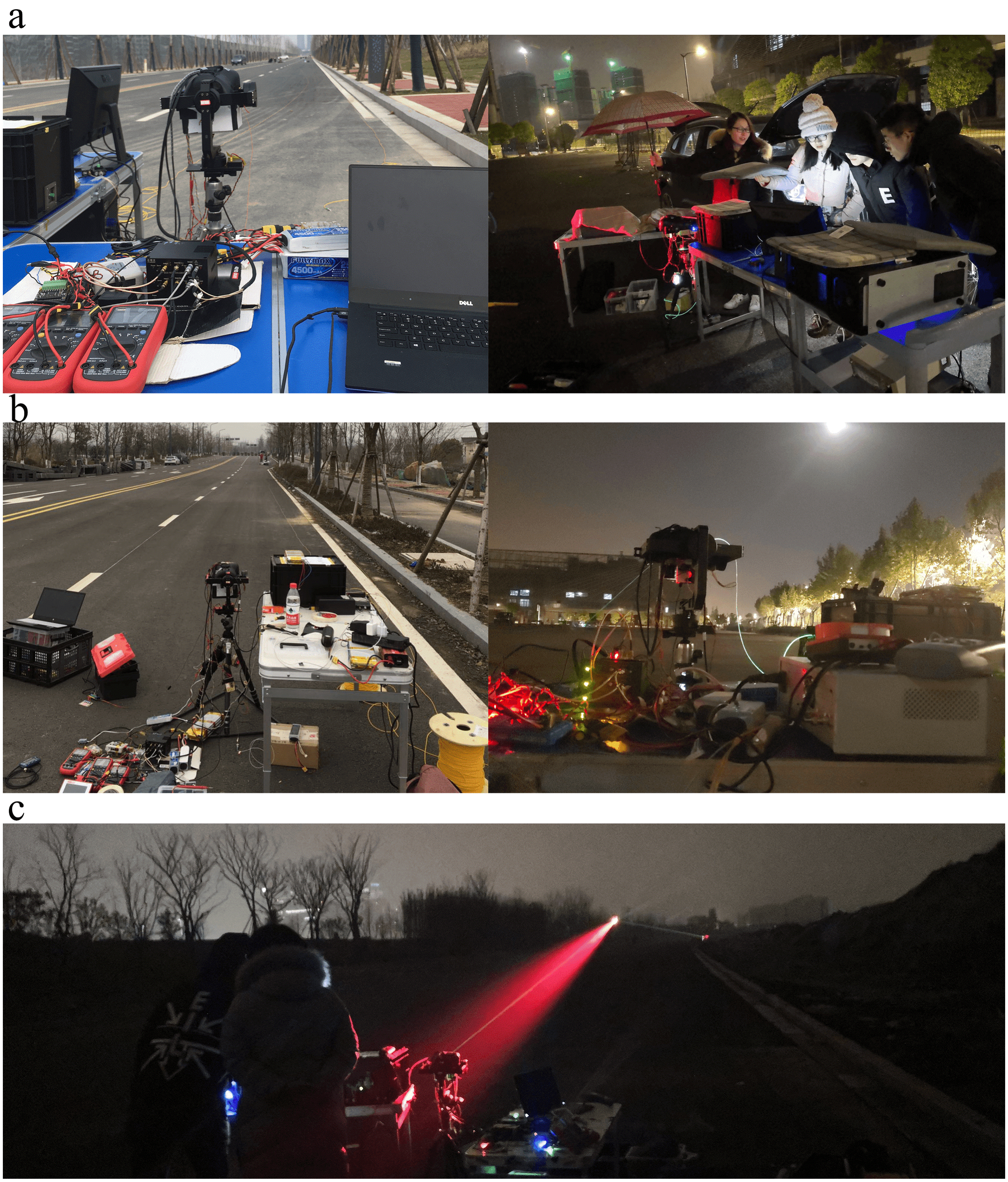

Supplementary Figure S5 | Alice and Bob ground station at daytime, clear night and rainy night. a, Alice station at daytime (left) and rainy night (right). **b,** Bob station at daytime (left) and clear night (right). **c**, A full view of the drone-based system at night.

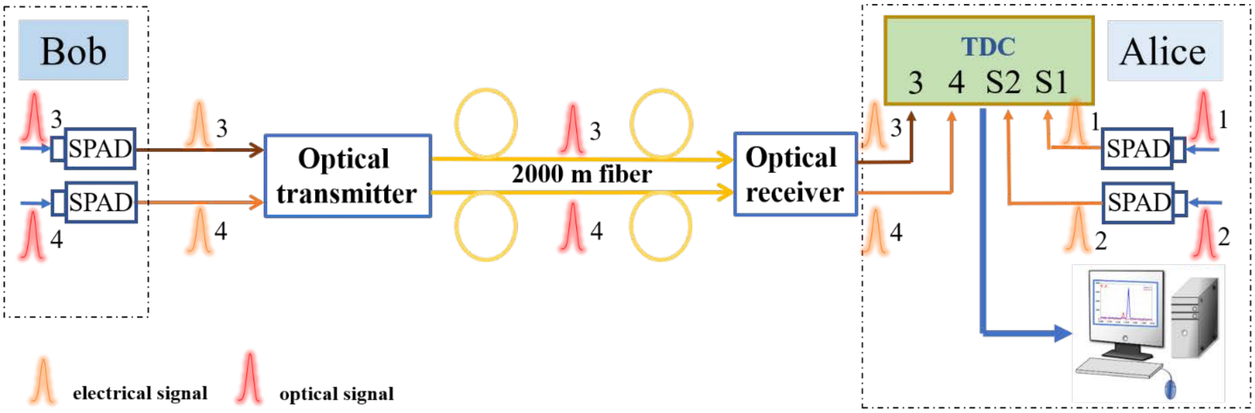

Supplementary Figure S6 | Classical communication link. Schematic of the classical communication system. The fiber channels transmit the photon clicks from Bob's SPADs to Alice station for coincident measurement. TCSPC1 processes the coincident events for SPADs pairs 1 & 3 and 1 & 4, while TDC processes the coincident events for SPADs pairs 2 & 3 and 2 & 4.

Supplementary References

1. Kato, K. & Takaoka, E. Sellmeier and thermo-optic dispersion formulas for KTP. *Appl. Optics* **41**, 5040-5044 (2002). doi: 10.1063/1.3590136

2. Bierlein, J. D. Potassium Titanyl Phosphate (KTP): Properties, Recent Advances And New Applications, *Proc. SPIE Int. Soc. Opt. Eng.* **1104**, 2-13 (1989). doi: 10.1117/12.960575
